# Instrumented gait characteristics and short-term changes after CAREN training in ischemic stroke: a pilot study

**DOI:** 10.3389/fpsyt.2026.1939460

**Published:** 2026-09-18

**Authors:** Qiaohui Zhu, Zhen Lyu, Shixing Ren, Xiaohong Qiao, Yabin Li, Haixia Feng

**Affiliations:** 1 Gansu Provincial Rehabilitation Center Hospital, Gansu, Lanzhou, China; 2 Gansu University of Chinese Medicine, School of Traditional Chinese and Western Medicine, Gansu, Lanzhou, China; 3 School of Psychology, Shanghai University of Sport, Shanghai, China

**Keywords:** CAREN, exploratory study, gait analysis, ischemic stroke, psychomotor function

## Abstract

**Background:**

Hemiplegic gait disturbance after ischemic stroke severely impairs patients’ mobility; however, existing tools still have limitations in both comprehensive evaluation and routine clinical application.

**Methods:**

This study enrolled 15 patients with ischemic stroke and 15 healthy controls, and collected gait parameters using the CAREN system. Among these participants, 5 patients completed a 5-day continuous training regimen and participated in a longitudinal before-after comparison analysis.

**Results:**

Baseline measures revealed pathological gait patterns. Post-intervention, step width, stride symmetry, and moment profiles showed short-term improvement trends.

**Conclusion:**

The CAREN system can objectively quantify gait abnormalities in stroke patients and capture short-term change trends, providing a reproducible instrumental gait assessment framework. This study is exploratory in nature, its independent efficacy requires validation through large-sample randomized controlled trials.

**Clinical Trial Registration:**

http://www.chictr.org.cn, identifier ChiCTR2600129541.

## Introduction

Psychomotor function serves as a crucial integrative link between cognition and motor execution (1–3), reflecting an individual’s coordination abilities in information processing, action planning, and motor output (4–6). Previous studies have demonstrated that psychomotor speed, motor initiation, and motor coordination can serve as behavioral markers of impaired central nervous system function (7, 8). However, such functions often cannot be directly and precisely quantified using conventional methods in routine clinical examinations. Gait, as one of the most common motor behaviors observed during daily activities, can reflect aspects of motor control, postural stability, bilateral coordination, and the efficiency with which walking tasks are performed to a certain extent; consequently, it provides a quantifiable behavioral window for observing functional changes following stroke (9, 10).

Ischemic stroke is one of the common causes of long-term motor impairments and restricted mobility (11, 12), and approximately 50%-70% of survivors experience lasting walking impairments (13, 14). Traditional rehabilitation has largely attributed post-stroke motor deficits to corticospinal tract damage and consequent muscle weakness, leading to interventions centered on strength training (15). However, numerous clinical observations have demonstrated that some patients with favorable muscle strength recovery still exhibit significant gait quality impairments (16–18)—including shortened stride length, increased stride width, weight-bearing asymmetry, and altered movement rhythm. This suggests that purely peripheral factors cannot fully account for all manifestations of post-stroke gait disturbances; rather, central-level factors—such as alterations in sensorimotor integration, motor planning, and bilateral coordination—may also be involved (19–23).

Current rehabilitation assessment systems face two major shortcomings. On the motor side, tools such as the Fugl-Meyer scale and Barthel Index capture gross functional status but fail to quantify subtle gait rhythm, initiation delay, or bilateral coordination (24–26). On the cognitive side, evaluations rely heavily on subjective questionnaires and interviews, which are susceptible to patient compliance and examiner bias (27, 28). These limitations make it challenging to objectively distinguish between the distinct contributions of peripheral motor disorders and central cognitive-motor integration impairments to gait abnormalities in clinical practice, thereby hindering detailed characterization and mechanistic exploration of post-stroke gait disturbances. Consequently, developing an objective assessment method that enables a more nuanced characterization of post-stroke gait abnormalities is of substantial practical importance.

The Computer-Assisted Rehabilitation Environment (CAREN) integrates immersive virtual environments, high-precision 3D motion capture, force plates, and real-time biofeedback, achieving millisecond temporal and millimeter spatial resolution in gait analysis (29, 30). It enables precise quantification of gait rhythm, spatiotemporal symmetry, center-of-mass control, and movement initiation efficiency, thereby addressing the deficiencies of traditional scales (31). Although CAREN has been applied to quantify pathological gait in Parkinson’s disease and cerebellar ataxia (32–35). Systematic evidence in ischemic stroke survivors is scarce, and a direct link between refined gait metrics and central functional recovery has not been established.

In this study, we used the CAREN system to conduct a quantitative analysis of gait characteristics in patients with ischemic stroke and healthy controls, and further observed gait changes in selected patients after short-term CAREN training. The aims of this study were to characterize gait patterns in stroke patients and preliminarily assess short-term changes in gait parameters following CAREN-based training, so as to lay a foundation for subsequent research with larger sample sizes that integrates clinical outcomes and neurobiological biomarkers.

## Materials and methods

This study involves human subjects and has been approved by the Ethics Committee of Gansu Province Rehabilitation Center Hospital (approval number: gsskfzxyy-ll-xm-2024004). All participants have signed a written informed consent form, clearly understanding the research purpose, procedures, and potential risks, and have voluntarily participated in this study. The subjects have signed a written informed consent form, granting permission for their imaging data to be used in the academic publication of this study.

### Study population

15 ischemic stroke patients (4 females, 11 males; Age: 50.73 ± 11.56 years) during their rehabilitation treatment were recruited at the New District of Gansu Provincial Rehabilitation Center Hospital and participated in 5-day gait training program. Based on the predefined screening criteria—full compliance with the 5 consecutive days of training, no adverse events or mid-trial dropouts, and complete gait measurement data—only 5 subjects were ultimately included for a longitudinal self-controlled before-and-after analysis. 15 healthy controls (10 females, 5 males; age: 56.8 ± 6.22 years) were enrolled in this study, and their gait data were collected to serve as baseline reference data (**Supplementary Figure 1**). Detailed clinical characteristics of the patients are provided in **Supplementary Table 1**.

All patients were enrolled with the following inclusion criteria: 1) clinically confirmed ischemic stroke with right hemispheric lesion causing left-sided motor dysfunction; 2) aged 18–70 years; 3) hemiplegic lower extremity at Brunnstrom stage III-V; 4) standing balance capacity graded 1-3; 5) voluntary participation with signed informed consent. Exclusion criteria were: 1) body weight outside system tolerance limits (< 20 kg or > 135 kg); 2) spine instability or fracture; 3) open skin lesions or thromboses in harness contact areas; 4) severe cardiovascular comorbidities or functional impairments. For the healthy group, inclusion criteria were aged 18–70 years, no clinically significant gait abnormalities, and voluntary participation with signed informed consent. Exclusion criteria were the same as for the patients.

### Assessment procedures and equipment

All gait assessments were conducted using the CAREN system, which integrates 18 motion-capture cameras (100 Hz), a dual-belt treadmill (Force Plate 1000Hz), and a 330-inch virtual reality screen. Participants wore a compression garment, and 22 reflective markers were placed on bilateral lower limbs and key anatomical landmarks of the pelvis according to the Human Body Model2 (HBM–II) model. Before formal data collection began, participants walked on the treadmill for approximately 2 minutes to acclimatize to the environment. Following this acclimatization period, 90 seconds of continuous gait data were collected at different speeds. For healthy control participants, gait data were collected at three fixed speeds: 0.1 m/s, 0.2 m/s, and 0.35 m/s. For patients, baseline and pre–post training assessments were performed at 0.35 m/s, which was the highest speed tolerated by all patients. Both cross-sectional and longitudinal comparisons were performed at 0.35 m/s. Gait events were automatically identified based on the vertical force threshold measured by the force plate (≥20 N), which specifically marked foot strike and toe-off moments. For each walking speed, the mean parameter value was calculated by averaging 15 consecutive valid gait cycles for subsequent statistical analysis.

Primary outcome measures: 1) Spatiotemporal parameters included step width, step length, stride length, and unilateral lower limb stance phase proportion. Stance phase proportion was defined as the ratio of unilateral stance time to the total duration of a complete gait cycle (stance time plus swing time); 2) Dynamic parameters: knee joint flexion/extension torque and ankle joint plantarflexion/dorsiflexion torque. Joint torques were calculated using an inverse dynamics approach and expressed in N·m/kg after body weight normalization. Symbol conventions: knee extension is positive (+), while knee flexion is negative (-); ankle dorsiflexion is positive (+), whereas ankle plantarflexion is negative (-). All raw kinematic and dynamic data were processed using a 6 Hz second-order zero-phase Butterworth low-pass filter; short-term marker loss was interpolated via cubic spline interpolation, and severely invalid data points were excluded.

### Training interventions

All patients underwent training interventions using the CAREN system, consisting of one session per day for 30–40 minutes over a period of 5 consecutive days. The training tasks included: (1) walking along a designated path within a virtual environment while avoiding obstacles; (2) adjusting stride length and step width based on visual feedback to achieve target symmetry; (3) balance reaction training through anterior-posterior platform perturbations. Training speed was incrementally increased based on the patient’s daily performance (starting at 0.1 m/s, with increments of ≤0.05 m/s, not exceeding a maximum of 0.5 m/s). Platform tilt angles and perturbation magnitudes were dynamically adjusted by the therapist according to the patient’s balance capacity to maintain a task success rate of approximately 60%-70%. The system provided real-time visual and auditory biofeedback (e.g., gait symmetry bar graphs, joint moment waveforms) during training to encourage active regulation of gait patterns. Patients wore safety harnesses throughout the session and were monitored by an assisting researcher to prevent falls (specific details are provided in **Table 1**).

**Table 1:** Description of CAREN scenarios and exercises used to train IS patients.

VR scenario	Description	Exercise goal
Rope bridge	The subject will walk across a bridge. The force plate adjusts to the bridge’s slope, and the sway aligns with its lateral movement. While crossing, the subject will face both uphill and downhill sections and must avoid birds. Such as birds appearing on the right side, requiring the subject to walk on the left side of the bridge.	To perform lower limb training through ascending and descending slopes and left-right stepping movements.
Step on it	The subjects walked continuously on a straight road scene viewed from above, with the screen displaying real-time visual feedback of footprints.	To adjust step width and stride length based on environmental cues to modulate step frequency, thereby conducting stride length and stride amplitude control training.
Perturb	In this scenario, balls will be ejected from the front, back, left, and right vents to disrupt the platform, requiring participants to maintain balance as much as possible.	Through responding to external disturbances, perform dynamic balance training.

### Statistical measurements

We used multiple statistical measurement metrics to conduct a comprehensive analysis and evaluation of the data. Data normality was assessed with the Shapiro-Wilk test. For cross-sectional intergroup comparisons: when data satisfied the assumptions of normal distribution and homogeneity of variances, the independent samples t-test was used; otherwise, the Mann–Whitney U test was applied. For longitudinal pre-post comparisons: the paired samples t-test was adopted; if the normality assumption was not met, the Wilcoxon signed-rank test was utilized. Due to the small sample size of the longitudinal subgroups (n=5), statistical inferences from this part should be interpreted with caution, and the results mainly focus on descriptive trends. The significance level was set at α = 0.05, and all tests were two-tailed. Continuous variables are reported as mean ± standard deviation (M ± SD), and categorical variables are presented as number of cases (percentage).

## Results

This study utilized the CAREN system, a cutting-edge neurorehabilitation platform. The system’s D-flow software synchronizes real-time data collection and performs preliminary data analysis (**Figure 1**).

**Figure 1:**
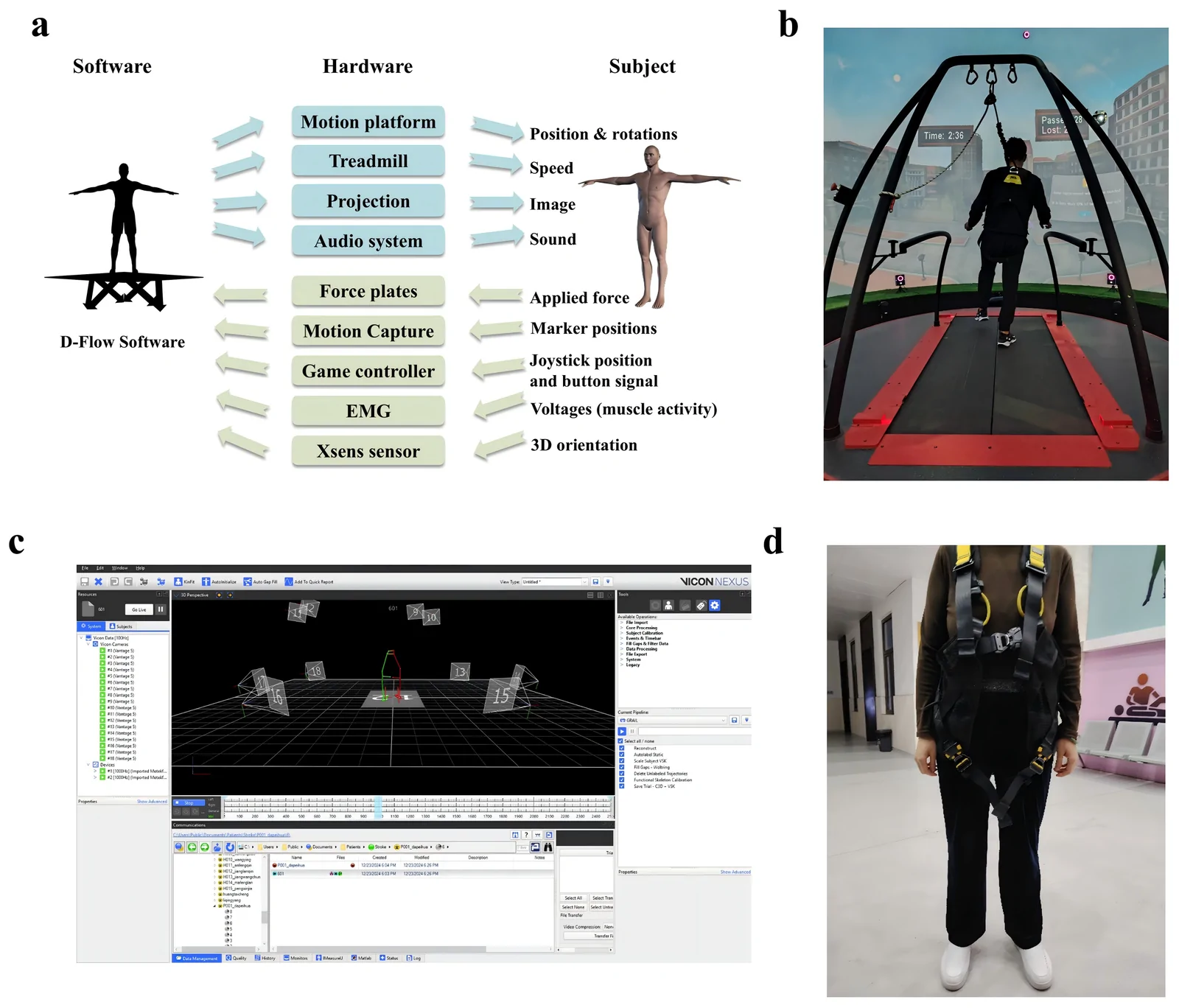
The illustration of the CAREN system. **(a)** CAREN’s workflow; **(b)** Patient walking inside the device; **(c)** CAREN software interface screenshot; **(d)** Patient in the equipped device.

We first examined the gait characteristics of healthy individuals. Using the CAREN system, we recorded the participants’ behavior patterns at different walking speeds (**Supplementary Figure 2**). The results showed that when walking at a fixed speed, healthy individuals exhibited stable and symmetrical gait parameters, balanced joint moment, and symmetrical loading. Additionally, they maintained this stability and efficiency when adjusting their walking speeds. Specifically, healthy individuals exhibited gait adaptability and stability (step width: 0.158 ± 0.042 m, step length: 0.253 ± 0.080 m, stride length: 0.494 ± 0.174 m; **Figure 2a**). The lower limb stance phase proportion show high symmetry (left 72.3% vs right 72.3%, Asymmetry Index = 0.03%; **Figure 2b**), which contributes to the maintenance of gait stability. In addition, we further observed distinct bilateral movement patterns, characterized by a combination of left knee flexion (-0.452 ± 0.876 N·m/kg) with right knee extension (1.423 ± 1.617 N·m/kg; **Figure 2c**). Concurrently, left ankle dorsiflexion (1.445 ± 1.830 N·m/kg) with right ankle plantarflexion (-3.510 ± 3.811 N·m/kg; **Figure 2d**). These moments collectively ensure the stability and propulsive efficiency of gait.

**Figure 2:**
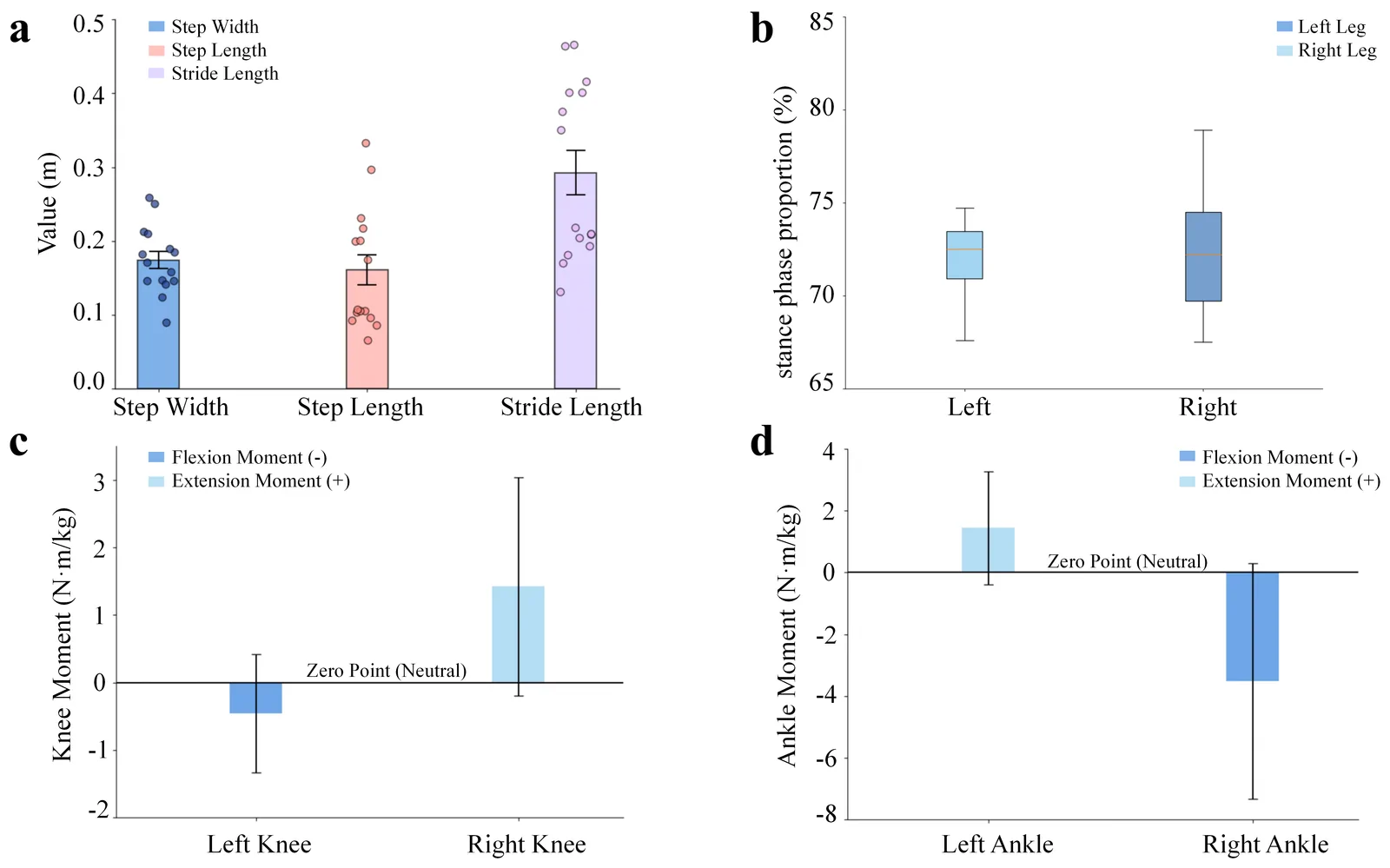
Gait characteristics of healthy individuals. **(a)** General gait parameters of healthy individuals; **(b)** Stance phase proportion between left and right legs; **(c)** Knee flex moment; **(d)** Ankle flex moment.

In contrast to healthy controls, stroke patients exhibited marked gait abnormalities (**Figure 3a**). Specifically, step and stride length were significantly shortened (0.123 ± 0.039 m, Mann-Whitney U = 62.0, p = 0.013;0.247 ± 0.087 m, Mann-Whitney U = 70.0, p = 0.029, respectively), while step width increased (0.205 ± 0.050 m; independent-samples t-test, t(28) = -2.04, p = 0.048) to enhance dynamic stability (**Figure 3b**). Concurrently, support time on the affected limb was significantly shorter than that on the unaffected side (76.0% ± 7.4% vs 80.9% ± 5.1%; paired-samples t-test, t(14) = 3.730, p = 0.002, **Figure 3c**), with an Asymmetry Index of 6.25%, reflecting asymmetric weight-bearing and increased reliance on the unaffected limb for balance. Joint moment patterns were similarly disrupted. The flexion moment of the affected knee joint was reduced (absolute value decreased from 0.452 to 0.244 N·m/kg; **Figure 3d**) during toe-off, thereby impairing propulsion. Additionally, the contralateral propulsion capacity was also below normal levels, manifested as a decrease in the absolute value of the right ankle moment from 3.510 to 0.627 N·m/kg (independent-samples t-test, t(28) = -2.585, p = 0.043, **Figure 3e**). Collectively, these findings suggest that stroke patients may tend to adopt adaptive gait patterns to maintain real-time dynamic stability.

**Figure 3:**
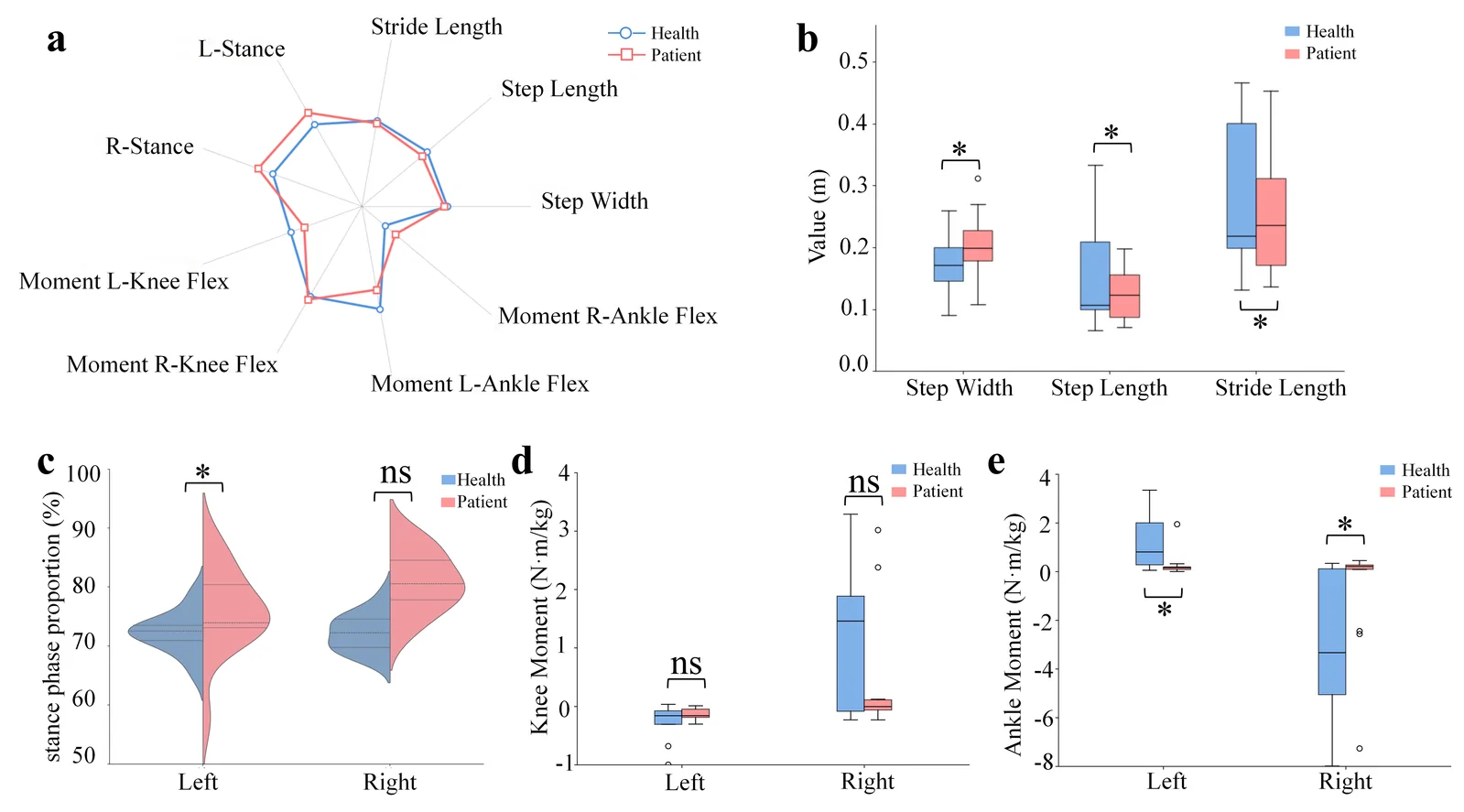
Comparison of gait characteristics between healthy individuals and stroke patients. **(a)** All gait parameters; **(b)** General gait parameters; **(c)** Stance phase proportion between health and patient; **(d)** Knee flex joint moment comparison; **(e)** Ankle flex joint moment comparison. Statistical difference significance is reported as *p< 0.05. Health, healthy individuals; Patient, stroke patient.

We further used the CAREN system to evaluate short-term changes in gait parameters following a 5-day CAREN training. Our results showed that patients exhibited significant improvement in both gait and biomechanical parameters (**Figure 4a**), with step width decreased from 0.204 m to 0.158 m (paired-samples t-test, t(4) = 3.609, p = 0.023), while step increased to 0.195 m (t(4) = -4.288, p = 0.013) and stride length increased to 0.329 m (t(4) = -3.493, p = 0.025), indicating enhanced stability and propulsion. Limb support symmetry also improved markedly, with the inter-limb difference in stance phase proportion narrowing from 4.9% to 2.3% (paired-samples t-test, t(4) = 2.933, p = 0.043; **Figure 4b**), reflecting more balanced weight-bearing. At the joint moment level, the unaffected-side knee flexion moment decreased from 0.753 to -0.022 N·m/kg, suggesting reduced compensatory loading (paired-samples t-test, t(4) = 2.894, p = 0.044; **Figure 4c**). Concurrently, the affected-side ankle dorsiflexion moment increased from 0.256 to 0.421 N·m/kg (paired-samples t-test, t(4) = -2.879, p = 0.044; **Figure 4d**), suggesting improved joint moment distribution on the impaired side. In summary, the observed gait changes following 5 days of training exhibited a consistent direction (**Figure 4e**): reduced step width, improved bilateral support symmetry, and a trend toward healthy control levels in ankle joint moment on the affected side. These preliminary observations suggest that short-term training based on CAREN may be associated with short-term improvement trends in gait spatiotemporal parameters and joint load distribution; however, its clinical significance and sustainability require further validation.

**Figure 4:**
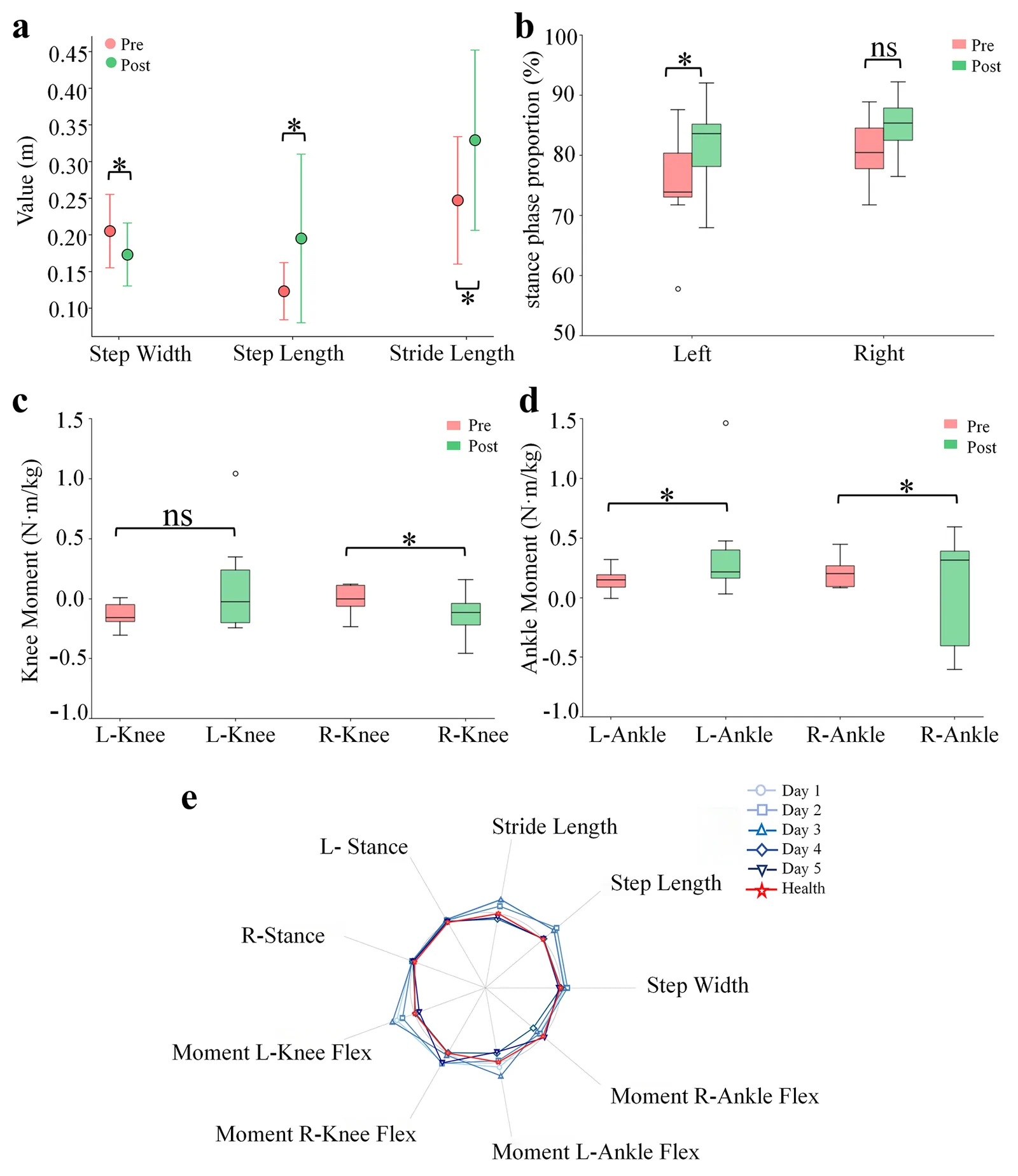
Comparison of stroke patients before and after treatment. **(a)** General gait parameters; **(b)** Stance phase proportion between pre and post; **(c)** Knee flex joint moment comparison; **(d)** Ankle flex joint moment comparison; **(e)** Gait parameters at different time points and in the healthy control group. Statistical difference significance is reported as *p< 0.05. Pre, before treatment; Post, after treatment.

## Discussions

This study employed a design combining cross-sectional comparison with longitudinal intervention. It quantitatively established a baseline biomechanical profile of steady-state gait in healthy individuals and identified characteristic pathological gait patterns in patients with ischemic stroke, which are primarily characterized by shortened step length, widened base of support, asymmetric limb loading, and compensatory joint moment patterns. After a five-day standardized CAREN intervention, patients showed short-term changes in spatiotemporal gait parameters, bilateral limb load symmetry, and joint moment distribution. These findings suggest that instrumental gait analysis can help detect subtle functional changes throughout the rehabilitation process.

These findings are largely consistent with previous studies on post-stroke gait impairments—post-stroke gait abnormalities typically manifest as shortened stride length, increased stride width, reduced stance phase proportion time, and asymmetric weight-bearing between the left and right limbs (36). From a psychomotor perspective, the disruptions in spatiotemporal gait symmetry and compensatory joint moment imbalance observed in this study can serve as potential external behavioral phenotypes, indicating impaired central cognitive–motor integration function, for exploratory analysis. However, limited by the study design, this research did not adopt standardized psychomotor or cognitive assessment tools. Accordingly, the association between gait characteristics and central integration function was only indirectly inferred through biomechanical indicators, and the direct causal relationship between the two factors still requires further verification by future studies that use cognitive scales.

Compared with traditional clinical scales, instrumental gait analysis can quantify these changes at a more granular spatiotemporal and biomechanical level, thereby providing objective information that is difficult to capture through bedside assessments. The CAREN system demonstrates its methodological value precisely at this level: it is capable of synchronously collecting kinematic and dynamic data under controlled conditions, and provides subtle abnormality indicators that are difficult to detect using traditional functional scales, such as the precise differences in bilateral support asymmetry and the quantitative changes in joint moment (37, 38). This addresses the limitations of conventional subjective scales, wearable sensors, and traditional motion capture technologies in terms of precision and synchronization (39–41). Therefore, CAREN is more suitable as a refined assessment platform, and serves as a supplement to routine rehabilitation assessments in clinical practice by providing objective and reproducible gait metrics.

This study also has several limitations. First, it did not incorporate standardized clinical outcome measures, making it impossible to determine whether the findings hold clinical significance. Second, the longitudinal analysis involved a relatively small sample size, so its results should be interpreted as exploratory observations only. Third, the longitudinal analysis lacked a concurrent control group, which precluded distinguishing between the relative contributions of CAREN training, spontaneous recovery, the familiarity effect, the sustained effects of concurrent conventional rehabilitation, and repeated-measures learning. Fourth, the intervention period was short, and long-term follow-up was lacking, meaning we could not assess the sustainability of the training effects or the long-term functional benefits obtained. Fifth, during the baseline data collection phase, this study did not systematically record demographic information including height, weight, and body mass index (BMI), which constitutes a gap in the data collection process. In addition, there was a discrepancy in gender distribution between the patient group and the healthy control group. Although this study did not explicitly require “gender matching”, this imbalance may potentially affect intergroup comparisons of gait parameters. In summary, the findings of this study should be regarded as exploratory. Future studies should expand the sample size, incorporate clinically meaningful outcome measures, and establish a randomized controlled group to validate the independent efficacy of CAREN training.

In conclusion, this study preliminarily demonstrates that the CAREN system can objectively quantify gait abnormality characteristics in patients with ischemic stroke, and can capture trends in certain gait parameters after short-term training. In addition, this study establishes a repeatable, instrument-based gait assessment framework under controlled conditions, and preliminarily verifies the operational feasibility of this framework for stroke rehabilitation assessment. Whether observed gait improvements reflect actual substantive recovery of psychomotor function still needs to be explored in future large-sample randomized controlled trials that integrate neuroimaging and standardized cognitive assessments.

## Data Availability

The raw data supporting the conclusions of this article will be made available by the authors, without undue reservation.
