# Supplementary material for: Instrumented gait characteristics and short-term changes after CAREN training in ischemic stroke: a pilot study
Source: Front Psychiatry. 2026 Sep 18;17:1939460. doi: 10.3389/fpsyt.2026.1939460 (PMC13630765; doi:10.3389/fpsyt.2026.1939460)
Supplement: Supplementary file 1 [file SupplementaryFile1.docx]

**Supplementary Materials**

**Supplementary Figure 1 Flowchart and details for the recruitment and enrollment process**

**Supplementary Figure 2 Gait parameters of healthy individuals at different speeds.** a. General gait parameters; b. Stance phase proportion between left and right legs; c. Knee flex joint moment comparison; d. Ankle flex joint moment comparison.

| ID | Sex | Age | Stroke duration(months) | Lesion side | Paretic side | Brunnstrom stage | Standing balance grade | Assistive device | Concurrent rehabilitation |
| --- | --- | --- | --- | --- | --- | --- | --- | --- | --- |
| P001* | F | 53 | 6 | Right | Left | Ⅲ | 2 | NONE | PT+OT |
| P002* | F | 52 | 5 | Right | Left | Ⅲ | 2 | NONE | PT+OT |
| P003* | M | 64 | 8 | Right | Left | Ⅳ | 3 | NONE | PT+OT |
| P004 | M | 59 | 7 | Right | Left | Ⅲ | 2 | NONE | PT+OT |
| P005 | M | 52 | 12 | Right | Left | Ⅲ | 2 | NONE | PT+OT |
| P006* | M | 59 | 8 | Right | Left | Ⅲ | 2 | NONE | PT+OT |
| P007 | M | 51 | 6 | Right | Left | Ⅳ | 2 | NONE | PT+OT |
| P008 | M | 51 | 9 | Right | Left | Ⅳ | 3 | NONE | PT+OT |
| P009 | M | 38 | 10 | Right | Left | Ⅳ | 2 | NONE | PT+OT |
| P010 | M | 33 | 5 | Right | Left | Ⅲ | 2 | NONE | PT+OT |
| P011 | M | 70 | 7 | Right | Left | Ⅲ | 2 | NONE | PT+OT |
| P012 | F | 60 | 5 | Right | Left | Ⅲ | 2 | NONE | PT+OT |
| P013* | M | 27 | 9 | Right | Left | Ⅳ | 2 | AFO | PT+OT |
| P014 | M | 48 | 8 | Right | Left | Ⅳ | 2 | NONE | PT+OT |
| P015 | F | 44 | 11 | Right | Left | Ⅳ | 3 | NONE | PT+OT |

**Supplementary Table 1 Demographic and clinical characteristics of 15 stroke patients:** *Patients marked with an asterisk completed the 5‑day CAREN training and were included in the longitudinal pre‑post analysis. Abbreviations: M, male; F, female; AFO, ankle‑foot orthosis; PT, physical therapy; OT, occupational therapy.

| parameters | 0.1m/s | 0.2m/s | 0.35m/s |
| --- | --- | --- | --- |
| Step.Width (m) | 0.174 ± 0.046 | 0.165 ± 0.036 | 0.158 ± 0.042 |
| Step.Length (m) | 0.161 ± 0.082 | 0.186 ± 0.055 | 0.253 ± 0.080 |
| Stride.Length (m) | 0.293 ± 0.119 | 0.364 ± 0.100 | 0.494 ± 0.174 |
| L-Stance phase proportion(%) | 79.6 ± 5 | 76.0 ± 4.1 | 72.3 ± 3.5 |
| R-Stance phase proportion(%) | 80.6 ± 5.5 | 74.7 ± 3.4 | 72.3 ± 3.2 |

**Supplementary Table 2	Gait parameters of healthy individuals at different speeds**

| parameters | 0.1m/s | 0.2m/s | 0.35m/s |
| --- | --- | --- | --- |
| L-KneeFlex (N·m/kg) | -0.832 ± 0.695 | -0.610 ± 0.788 | -0.452 ± 0.876 |
| R-KneeFlex (N·m/kg) | 0.842 ± 1.030 | 1.124 ± 1.300 | 1.423 ± 1.617 |
| L-AnkleFlex (N·m/kg) | 2.279 ± 2.037 | 1.539 ± 1.546 | 1.445 ± 1.830 |
| R-AnkleFlex (N·m/kg) | -1.774 ± 1.880 | -2.604 ± 2.577 | -3.510 ± 3.811 |

**Supplementary Table 3 Dynamic parameters of healthy individuals at different speeds**
